# Migration, early axonogenesis, and Reelin-dependent layer-forming behavior of early/posterior-born Purkinje cells in the developing mouse lateral cerebellum

**DOI:** 10.1186/1749-8104-5-23

**Published:** 2010-09-01

**Authors:** Takaki Miyata, Yuichi Ono, Mayumi Okamoto, Makoto Masaoka, Akira Sakakibara, Ayano Kawaguchi, Mitsuhiro Hashimoto, Masaharu Ogawa

**Affiliations:** 1 Department of Anatomy and Cell Biology, Nagoya University Graduate School of Medicine, 65 Tsurumai-cho, Showa-ku, Nagoya, Aichi 466-8550, Japan; 2 Laboratory for Cell Culture Development, Brain Science Institute, RIKEN, 2-1 Hirosawa, Wako, Saitama 351-0198, Japan; 3 KAN Research Institute, Inc., Kobe MI R&D Center, 6-7-3 Minatojima-minamimachi, Chuo-ku, Kobe, Hyogo 650-0047, Japan; 4 Hoshimoto Research Unit, Brain Science Institute, RIKEN, 2-1 Hirosawa, Wako, Saitama 351-0198, Japan

## Abstract

**Background:**

Cerebellar corticogenesis begins with the assembly of Purkinje cells into the Purkinje plate (PP) by embryonic day 14.5 (E14.5) in mice. Although the dependence of PP formation on the secreted protein Reelin is well known and a prevailing model suggests that Purkinje cells migrate along the 'radial glial' fibers connecting the ventricular and pial surfaces, it is not clear how Purkinje cells behave in response to Reelin to initiate the PP. Furthermore, it is not known what nascent Purkinje cells look like *in vivo*. When and how Purkinje cells start axonogenesis must also be elucidated.

**Results:**

We show that Purkinje cells generated on E10.5 in the posterior periventricular region of the lateral cerebellum migrate tangentially, after only transiently migrating radially, towards the anterior, exhibiting an elongated morphology consistent with axonogenesis at E12.5. After their somata reach the outer/dorsal region by E13.5, they change 'posture' by E14.5 through remodeling of non-axon (dendrite-like) processes and a switchback-like mode of somal movement towards a superficial Reelin-rich zone, while their axon-like fibers remain relatively deep, which demarcates the somata-packed portion as a plate. In *reeler *cerebella, the early born posterior lateral Purkinje cells are initially normal during migration with anteriorly extended axon-like fibers until E13.5, but then fail to form the PP due to lack of the posture-change step.

**Conclusions:**

Previously unknown behaviors are revealed for a subset of Purkinje cells born early in the posteior lateral cerebellum: tangential migration; early axonogenesis; and Reelin-dependent reorientation initiating PP formation. This study provides a solid basis for further elucidation of Reelin's function and the mechanisms underlying the cerebellar corticogenesis, and will contribute to the understanding of how polarization of individual cells drives overall brain morphogenesis.

## Background

The cerebellum plays an essential role in the coordination of posture and locomotion, head and eye movements, and a wide range of motor activities. These functions depend on the structural organization of the cerebellar cortex, in which the Purkinje cells receive input from multiple sources in the central nervous system either directly or via parallel fibers of the granule cells [1-3]. Purkinje cells are generated during the early embryonic period from the ventricular zone (VZ) facing the fourth ventricle [4,5] and migrate outward towards the pial side to subsequently form a monolayer (Purkinje cell layer) during the early postnatal days [6-10]. Just superficial to the perinatal Purkinje cell layer, there is a transient layer called the external granular layer (EGL) consisting of both differentiating granule neurons and their precursor cells. EGL precursors require Sonic hedgehog, which is supplied by the underlying Purkinje cells, to expand the granule neuron population postnatally [11]. Thus, the arrangement of the Purkinje cells during embryonic development is a key histogenetic event and consequently determines the overall cerebellar volume, the foliation pattern, and the intensity of the Purkinje-granule association, a lifeline of the cerebellar circuitry.

How this arrangement of Purkinje cells is established is only partly understood. In the cerebellum of *reeler *mice, Purkinje cells cannot form a normal layer under the pial surface and instead are clustered near the deep nuclear (DN) neurons [12]. Reelin, an extracellular glycoprotein encoded by the *reelin *gene mutated in *reeler *mice [13-15], begins to be expressed near the pial surface on embryonic day 13.5 (E13.5) by prospective DN neurons [16-18]. At E13.5, these DN neurons are still superficially migrating towards the anterior side from the posterior end of the cerebellum, the rhombic lip (RL) [18-20]. One day later (at E14.5), the arrangement of Purkinje cells into a structure several cells thick (called the Purkinje plate (PP)) is observed in normal cerebella; in *reeler *cerebella, however, abnormal distribution of Purkinje cells (lack of the PP) is clearly seen [7,21]. The PP is formed just beneath a Reelin-rich zone to which RL-derived cells are continuously supplied; DN neurons, the first producers of Reelin, are followed by EGL cells [16,17]. This *in vivo *spatial relationship between the Reelin-rich zone and Purkinje cells has been reproduced in experimental manipulations of Reelin-producing zones by explant culture and *in utero *transplantation [22], suggesting that Reelin may somehow promote or instruct nascent Purkinje cells to take a position close to the Reelin-rich zone. However, our understanding of the cellular scenarios involved in the initiation of PP formation at E14.5 is very limited. Furthermore, it is not known what nascent Purkinje cells look like *in vivo*.

To elucidate PP formation, there are three specific issues, each of which should be addressed with single-cell resolution studies. First, although a prevailing model suggests that Purkinje cells migrate along the 'radial glial' fibers connecting the ventricular and pial surfaces [6,10,23-26], whether or not this model fits with the actual morphology and behavior of Purkinje cells in the formation of the PP should be verified. Second, the key cellular behaviors that initiate or fail to initiate the PP in the presence or absence of Reelin need to be established. Third, whether the migratory and positioning behavior of Purkinje cells is linked with their polarization towards circuit formation should be determined. The time frame and manner in which Purkinje cells begin axonogenesis should also be examined.

By using an adenovirus-mediated pulse-chase method [27,28] in combination with recently established Purkinje-cell-specific markers [26,29,30] and slice culture [31], the present study focused on the developing mouse lateral cerebellum until E14.5 and unveiled three important behaviors of a subset of Purkinje cells born at E10.5 in the posterior region: tangential migration (at approximately E13.5); early axonogenesis (at approximately E12.5); and Reelin-dependent 'posture' change initiating PP formation (between E13.5 and E14.5).

## Results

### Purkinje cells that initiate PP formation at E14.5 are born on E10.5 in a posterior VZ

**Figure 1:**
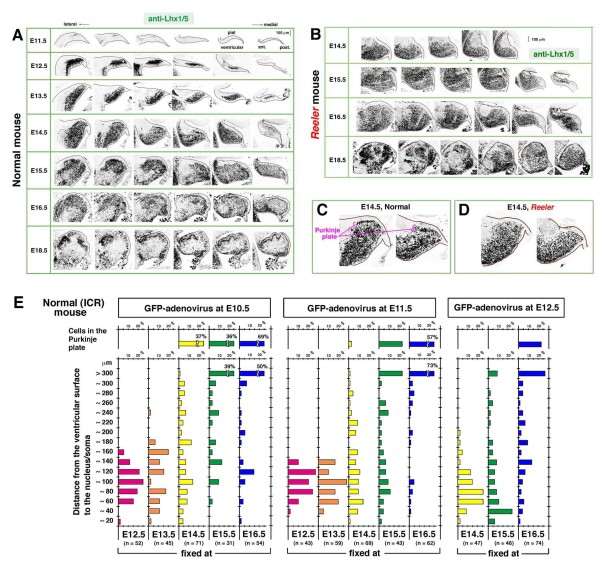
**Stage-dependent changes of the overall distribution of Purkinje cells in embryonic cerebella demonstrated with Lhx1/5 immunohistochemistry and adenovirus-mediated pulse-chase methods**. (A-D) Anti-Lhx1/5 immunostained sagittal sections of normal (ICR or +/rl) (A, C) and *reeler *(rl/rl) (B, D) cerebella. Sections in each row of (A, B) are arranged in a lateral-to-medial order. The dorsal side is on the top of each picture and the posterior side is on the right. At E14.5, the Purkinje plate (PP) emerges in the normal (C) but not in *reeler *(D) cerebellum, and this initial difference became progressively enhanced until the perinatal period (E18.5), when the subpial layering versus deeply clustered patterns became complete. **(E) **Histogram showing the distance of green fluorescent protein (GFP) and Lhx1/5 double positive cells from the ventricular surface at the indicated stages (bottom) following Lyn-Venus adenovirus injections at E10.5, E11.5, and E12.5. Consistent with a general tendency that cells born earlier migrate further, the cells that contributed best to the emergence of the PP at E14.5 were born on E10.5 (presented separately at the top of the graph). Note, however, that some of the E10.5-born cells are seen deeply (within 150 μm from the ventricular surface) even at E15.5 and E16.5.

As the first step in elucidating the mechanism of the emergence of the PP, we analyzed the identity of cells incorporated into the initial PP. Adenoviral labeling is an effective tool for tracing the behavior of a cohort of neurons born on the day of virus injection [27,28]. We sought to determine whether an anti-Lhx1/5 antibody, a marker for nascent Purkinje cells [26,29], is useful throughout embryonic development and also in *reeler *cerebella. In the normal cerebellum at E18.5, anti-Lhx1/5 distinguished a subpial cellular alignment typical of Purkinje cells that has previously been identified using different markers such as calbindin [8,16]. This perinatal Lhx1/5 immunoreactivity was well traceable back to E11.5, soon after the earliest cohort of Purkinje cells is generated [4,5,8,27] (Figure 1A). In *reeler *cerebella, Lhx1/5 immunoreactivity was observed with similar intensity to that seen in normal cerebella, and the absence of the PP at E14.5, which was previously reported based on Nissl staining [7], was confirmed (Figure 1B-D).

Following *in utero *injection of adenovirus vectors carrying a membrane-targeted green fluorescent protein (GFP) derivative (lyn-Venus) [32,33] at E10.5, E11.5, or E12.5, the position of cells double immunostained with anti-GFP and anti-Lhx1/5 antibodies was analyzed in normal cerebella from E12.5 to E16.5 (Additional file 1). While 37% of the total GFP^+^Lhx1/5^+ ^cells obtained by injection at E10.5 and analyzed at E14.5 were found in a region corresponding to the PP, incorporation of later-born cells into the E14.5 PP was very limited (3% for E11.5-born cells) or not observed (E12.5-born cells), indicating that the Purkinje cells that initiate the PP at E14.5 are generated mainly at E10.5. Histograms for birthdate- and track time-dependent changes of the distance of GFP^+^Lhx1/5^+ ^cells from the ventricular surface show a general tendency that cells born earlier were seen further from the ventricular surface (Figure 1E). However, not all E10.5-born cells quickly moved towards the pial side to be integrated into the PP; some were still in a deep, periventricular region at E15.5 and E16.5. This result, together with the Lhx1/5 immunoreactivity results, is consistent with the possibility that Purkinje cells born in the VZ of the posterior cerebellar region may be more quickly incorporated into the PP than anterior-born cells. This interpretation was further supported by the observation of individual GFP^+^Lhx1/5^+ ^cells in normal cerebella at E12.5 and E13.5, as described below.

### Early/posterior-born Purkinje cells migrate tangentially in the lateral cerebellar primordium

**Figure 2:**
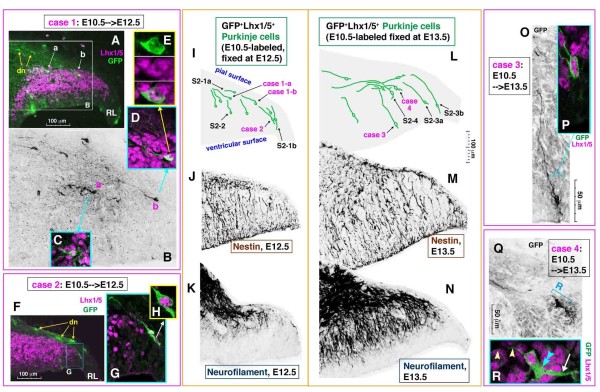
**The early/posterior-born Purkinje cells are elongated radially and tangentially in the lateral cerebellum at E12.5 and E13.5**. **(A-Q) **The morphology and orientation of the E10.5-adenovirally labeled nascent Purkinje cells in E12.5 (A-I) and E13.5 (L, O, Q) cerebella are compared with immunoreactivity for Nestin (J, M) (almost identical staining patterns were obtained by using RC2 (not shown)) and Neurofilament (K, N). dn, prospective deep nuclear neurons; RL, rhombic lip. In (I, L), traces of representative cases of E10.5-born nascent Purkinje cells (including cases 1a, 1b, 2, 3, and 4 in this figure, as well as those shown in Additional file 2) are summarized in an illustration of a 'standardized' cerebellar primordium at E12.5 or E13.5. The scale is common in panels (I-N). Note that E10.5-born Purkinje cells seen in an outer zone near the pial surface are tangentially elongated whereas those in deeper regions are more radially elongated. Neurofilament^+ ^radial fibers markedly increased between E12.5 and E13.5 and appear to outnumber Nestin^+ ^fibers at E13.5. **(R) **Each tangentially oriented Purkinje cell at E13.5 (Q, R) is polarized with a single thin and long process extended anteriorly (arrowheads) and a thick cytoplasmic part (double blue arrowhead) from which a few thick processes (arrow) are extended posteriorly.

**Figure 3:**
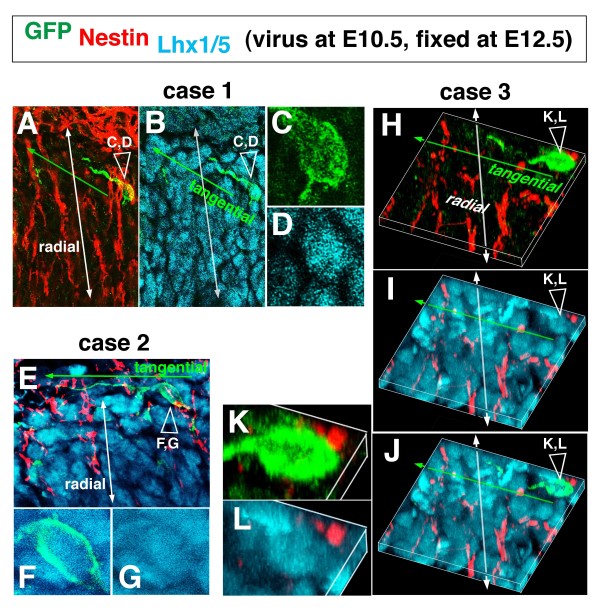
**Triple immunolabeling analysis to demonstrate the relationship between tangentially oriented nascent Purkinje cells and radial fibers**. **(A-G, K, L) **Sections as used for the E10.5 to E12.5 tracing analysis (Figure 2A-I) were stained with anti-GFP (green), anti-Lhx1/5 (cyan), and anti-Nestin (red). In each of the three representative cases, magnified views for the somal part (open arrowhead) are provided to confirm Lhx1/5 immunoreacivity. **(H-L) **In case 3, three-dimensional reconstructions are shown.

To understand how some of the E10.5-labeled nascent Purkinje cells contribute most quickly to PP formation, we analyzed the morphology and position of these cells in E12.5 and E13.5 cerebella. This analysis was important as a basis for elucidating the role of Reelin in the initiation of the PP at E14.5. PP formation was most clearly visible in a region of the lateral cerebellum at E14.5 (Figure 1A); thus, our analysis focused on this region. Adenoviral injection at E10.5 labeled both nascent Purkinje cells and the prospective DN neurons (Figure 2; Additional file 2A). The latter population originates from the posterior-most cerebellar region, the RL, and migrates tangentially along the pial surface, which has been demonstrated by fate-mapping studies [18-20]. Just below the tangentially migrating DN neurons (Lhx1/5-negative; cells labeled 'dn' in Figures 2A, F and Additional file 2A) in E12.5 cerebella, GFP^+^Lhx1/5^+ ^cells similarly elongated parallel to the pial surface were observed ('case 1a' and 'case 1b' cells in Figure 2A, B, I (magnified in 2C-E); cell 'a' in Additional file 2A). Based on their traceability, which was similar to that of migratory route back to the RL of the GFP-labeled DN neurons, the tangentially oriented GFP^+^Lhx1/5^+ ^cells were considered to have taken a similar (though less superficial) tangential migratory route from a posterior VZ next to the RL ('case 2' in Figure 2F-I; 'cell b' in Additional file 2A). Tangentially oriented GFP^+^Lhx1/5^+ ^cells were also abundant in E13.5 cerebella (Figure 2L). Evidently, these tangentially oriented Purkinje cells (Figure 2I, L) were not parallel to radially oriented fibers positive for Nestin (Figures 2J, M, and 3), indicating that they do not migrate radially.

**Figure 4:**
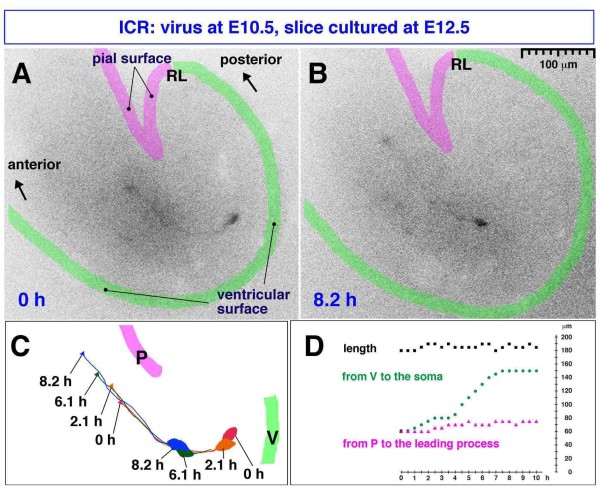
**Time-lapse monitored tangential migration of a Purkinje-like cell**. **(A, B) **A Venus-expressing E10.5-born Purkinje-like cell in an E12.5 cerebellar slice (see also Additional file 3). **(C) **Traces of migration: P, pial surface; V, ventricular surface; arrow, tip of the leading process. **(D) **Graph depicting the length of the observed cell, distance from the ventricular surface to the soma, and distance from the pial surface to the tip of leading process. Migration of this cell is characterized by radial movement of the soma away from the ventricular surface (green line) and tangential movement of the leading process parallel to the pial surface (magenta line). Note that this cell may have originated from a VZ region far (> 200 μm) from the rhombic lip (RL), and also that tangential movement of its leading process occurred at a depth 60 to 80 μm from the pial surface, together indicating that this cell was not a DN neuron. DN neurons originate from the RL and migrate very superficially (< 20 μm from the pial surface), as seen in cases 1 and 2 in Figure 2A, F and the case in Additional file 2A.

The posterior E10.5-born Lhx1/5^+ ^cells may take an almost entirely tangential migratory route. A slightly more anterior-born population of Lhx1/5^+ ^cells, which were initially radial ('case 3' in Figure 2O and a cell shown in Additional file 2B), also appeared to subsequently follow more tangential orientations (Figure 2I, L). As the somata of these Punkinje cells approached an outer region, their leading process extended anteriorly, parallel to the pial surface (Figure 2I, L). Time-lapse observation of an E10.5-born Purkinje-like cell in an E12.5 cerebellar slice demonstrated that radial migration (somal part) and tangential migration (leading process part) coincide in nascent Purkinje cells (Figure 4A-D; Additional file 3). Although immunostaining was not carried out in this time-lapse monitored case, we believe that this single GFP^+ ^cell labeled through viral injection at E10.5 was a Purkinje cell based on its morphology and behavior, which were consistent with our *in vivo *findings. Our separate quantification also shows that most GFP^+ ^cells within a deep cerebellar region sandwiched by the outermost territory for DN neurons and the VZ are Lhx1/5^+ ^(95% in the E10.5 to E12.5 analysis; n = 52/53).

### Purkinje cells begin axonogenesis at E12.5

**Figure 5:**
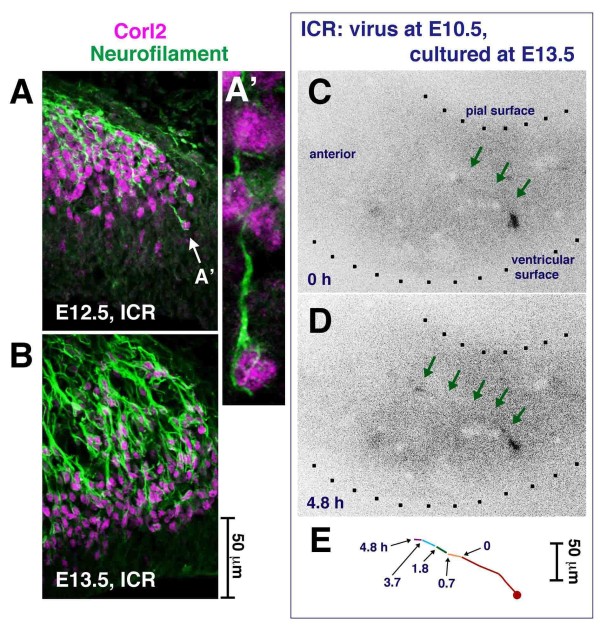
**Early axonogenesis in nascent Purkinje cells**. **(A, B) **Anti-Neurofilament and anti-Corl2 double immunostaining showing that many Purkinje cells have axon-like fibers running either radially or tangentially in E12.5 and E13.5 cerebella. **(C-E) **Time-lapse observation of an E10.5-born Purkinje-like cell extending an axon-like fiber (arrows) to the anterior side in an E13.5 cerebellar slice (see also Additional files 4B and 5). Although not immunostained, it is highly likely that the cell in this time-lapse is a Purkinje cell based on our *in vivo *data, which indicate that most GFP^+ ^cells within a deep cerebellar region sandwiched by the outermost territory for DN neurons and the VZ are Lhx1/5^+ ^(96% in the E10.5 to E13.5 analysis; n = 45/47).

The tangential orientation of the posterior E10.5-born Purkinje cells in an outer zone in lateral E12.5 to E13.5 cerebella (Figure 2I, L) was similar to that of fiber bundles immunoreactive for Neurofilament (NF; Figure 2K, N). In addition to the heavily accumulated tangentially oriented fibers, immunoreactive fibers, sparsely observed at E12.5 and significantly increased by E13.5, were observed running radially (Figure 2K, N). Corl2 is another nascent Purkinje cell marker whose expression almost overlaps with that of Lhx1/5 [30]. Cerebellar sections that were double immunostained against NF and Lhx1/5 or Corl2 were examined by confocal microscopy to determine whether these tangentially and radially oriented NF^+ ^fibers belonged to nascent Purkinje cells (Figure 5A, B; Additional file 4). Most Corl2^+ ^cells (71% (229/321 cells counted in the posterior half of the cerebellum at E12.5) and 85% (298/352 cells) at E13.5) were NF^+ ^(the NF expression rate was greater in the tangential part (almost 100%) than the radial part).

**Figure 6:**
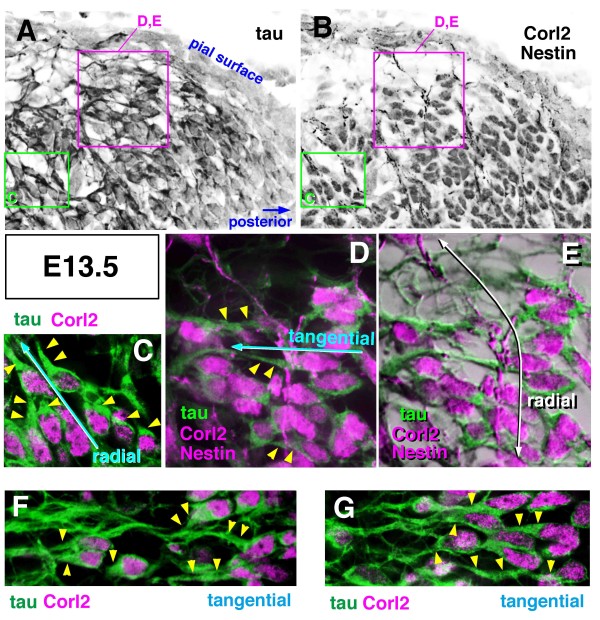
**Expression of tau protein in axon-like fibers of nascent Purkinje cells at E13.5**. **(A-G) **Cerebellar sections were first treated with anti-Corl2 (magenta in (C-G); nuclear) and anti-tau (green in (C-G)), and then stained with anti-Nestin (magenta in (D, E); fibrous); (C, F, G) show images before anti-Nestin immunostaining and (D, E) show images after it. Panels (C-E) show magnified views as indicated in (A, B). Panel (E) was made by superimposing colored fluorescent images on black-and-white images to increase the visibility of the tissue structure. Intense tau immunoreactivity was detected in a thickened proximal part (arrowheads) of the axon-like radial or tangential processes. Note that the tau^+ ^tangential fiber crosses the Nestin^+ ^radial fiber (D, E).

Immunostaining further revealed that Corl2^+ ^Purkinje cells expressed tau from E13.5 (Figure 6). At E12.5, they were negative for tau (data not shown). Tau immunoreactivity at E13.5 was similar to that for NF, intensely staining the fibers running anteriorly and radially (Figure 6). Addition or extension of new radially or anteriorly (tangentially) oriented fibers was observed live in slices containing E10.5-born GFP^+ ^cells (Figure 5C-E; Additional files 4B and 5). Afferent fibers derived from the vestibular ganglion are known to be the earliest among cerebellar afferents [34]. Thus, to determine whether these afferent fibers might also contribute to the radial NF^+ ^fibers within E12.5 cerebella, DiI was inserted into the vestibular ganglion. DiI^+ ^fibers were abundant in a superficial zone, but not observed in the deeper areas where NF^+ ^fibers run radially (n = 5 brainstem preparations; Additional file 4C).

Collectively, these results suggest that nascent Purkinje cells (not only posterior-born cells, but also more anterior-born ones) may start axonogenesis from E12.5 through the expression of NF and then tau. This timeline, axonogenesis from E12.5, is consistent with a recent suggestion by Joyner's group, using visualization of nascent Purkinje cells by a novel transgenic system (Pcp2-CreER-IRES-hAP), that axonogenesis may begin between E11 and E14 [35]. This interpretation for early axonogenesis is further supported by our observations towards E14.5, as described below.

### Migration and axonogenesis are normal in *reeler *cerebella until E13.5

**Figure 7:**
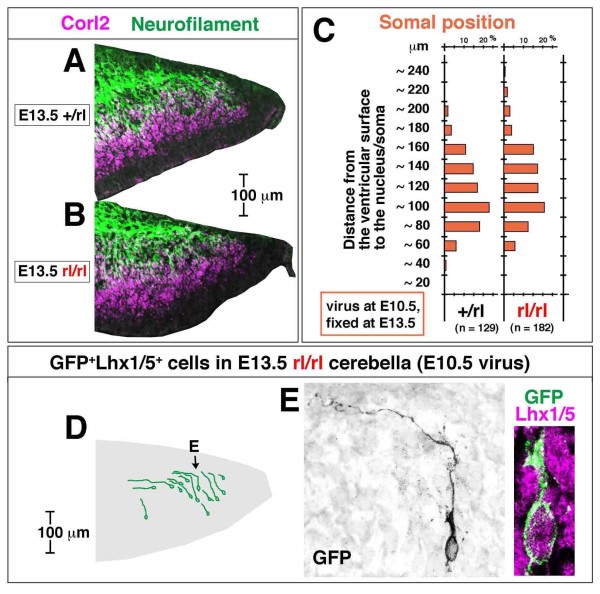
**Comparison of axonogenic and migratory behaviors of nascent Purkinje cells between normal and *reeler *cerebella at E13.5**. **(A-C) **The expression pattern of Corl2 and Neurofilament (A and B) and the position of E10.5-adenovirally labeled GFP^+ ^cells (C) are indistinguishable between normal (+/rl) and *reeler *(rl/rl) cerebella at E13.5 (each comparison was made between littermates). **(D, E) **Normal axonogenic behavior of E10.5-born Purkinje cells in *reeler *cerebella until E13.5 is also suggested by traces of individual GFP^+^Lhx1/5^+ ^cells. (E) An E10.5-born Purkinje cell observed in an E13.5 *reeler *cerebellar section. Note that this cell successfully migrated to an outer cerebellar region with an anteriorly extended axon-like fiber. Non-axon-like thick processes (as seen in the posterior or ventricular pole of Purkinje cells in normal E13.5 cerebella (Figure 2Q, R; Additional file 2D)) are not observed.

The above-mentioned radial and tangential somal migration and the extension of NF^+ ^fibers until E13.5 were normal in *reeler *cerebella (Figure 7). Corl2 and NF were expressed in *reeler *cerebellar sections at E12.5 (data not shown) and E13.5 (Figure 7A, B) in a spatiotemporal pattern that was indistinguishable from that observed in normal cerebella. Adenovirus-mediated pulse-chase examinations from E10.5 to E13.5 assessed the distance of the E10.5-born Purkinje cell somata from the ventricular surface. They revealed that the initial migration of E10.5-born Purkinje cells was normal (Figure 7C), with well-extended axon-like fibers (anteriorly and/or pially; Figure 7D, E). We observed that Purkinje cells in *reeler *cerebella at E13.5 exhibited less complicated morphology for non-axon-like processes (Figure 7D, E) than those in normal cerebella. Furthermore, the latter was more extensively extended posteriorly from the soma and branched (Figure 2L, Q; Additional file 2D). This might be a morphological sign that could potentially be related to or lead to the failure to form the PP by E14.5 in *reeler *mice (described below).

### Purkinje cells form a plate by changing their 'posture' between E13.5 and E14.5 in a Reelin-dependent manner through remodeling of dendrite-like processes

**Figure 8:**
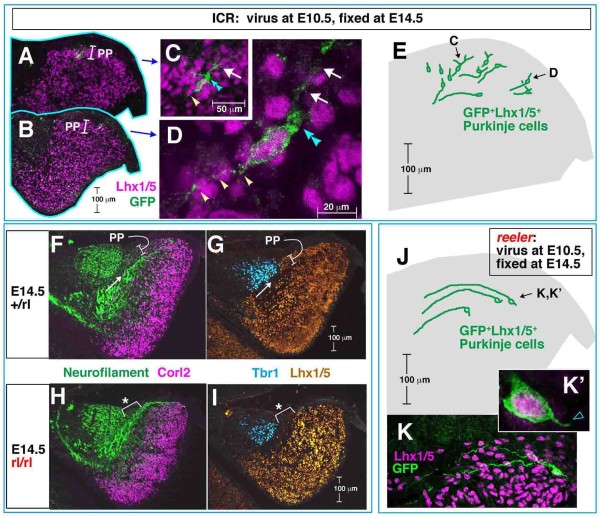
**Early/posterior-born Purkinje cells normally change their posture to initiate the formation of a plate between E13.5 and E14.5**. **(A-E) **Distribution and orientation of E10.5-born Purkinje cells in E14.5 normal (ICR mouse) cerebella. Traces of representative cases of Purkinje cells that were in an outer zone and oriented perpendicular to the pial surface (about 40% of the total E10.5-labeled Purkinje cells, including cells shown in (C, D)), mostly consisting of the Purkinje plate (PP), are summarized in an illustration of a standardized cerebellum. Note that a thin axon-like process (white arrowheads) is situated antero-ventricularly while a thick cytoplasmic part (double blue arrowheads) is seen postero-pially with extension of a few thick processes (arrows) towards the pial side (C, D). **(F-I) **Comparison of the relationship between Purkinje cell somata (stained with anti-Corl2 (F, H) and anti-Lhx1/5 (G, I)) and axon bundles (stained with anti-Neurofilament (F, H)) between normal (F, G) and *reeler *(H, I) cerebella at E14.5. Note that the PP in normal cerebella is demarcated by an axon bundle (arrow) running below. In *reeler *cerebella (n = 3 independent samples), the lack of PP (asterisked part) is associated with the persistent positioning of the axon bundle on the pial side. Tbr1 immunostaining suggests that DN neuron formation is normal in *reeler*. **(J-K') **None of the E10.5-born Purkinje cells adenovirally examined in E14.5 *reeler *cerebella (0/32 cells) showed the perpendicular orientation seen in normal cerebella. Three representative cases of cells that exhibited a horizontal orientation (13/32 cells) with a well-anteriorly extended axon-like process are traced. The remaining cells are more radially oriented, with axon-like fibers at the top, as frequently observed in E13.5 normal and *reeler *cerebella.

To ascertain how the PP normally emerges on E14.5, we first compared the morphologies of single Purkinje cells between E13.5 and E14.5. In normal cerebella at E14.5, Purkinje cells labeled by adenovirus injection at E10.5 exhibited an orientation different from that observed at E13.5. While their orientation in the outer/dorsal cerebellar region at E13.5 was tangential or parallel to the pial surface with anteriorly extended axon-like fibers (Figure 2L, N, Q; Additional file 2D), the orientation of cells comprising the PP in the outer/dorsal region at E14.5 was more perpendicular to the pial surface (Figure 8A-E). Each of the PP cells had short processes towards the pial side and its axon-like fiber was 'below' the soma (deeper from the pial surface than the soma). This interpretation of their morphology at E14.5 as 'axon-bottom' is consistent with previous suggestions from Golgi studies [1,7] and genetic fate-mapping [35].

Next, to examine how a Purkinje cell's orientation and polarization at the single cell level contribute to the overall structure of the PP, comparative immunostaining against NF, Corl2, and Lhx1/5 was carried out. In normal cerebella at E14.5, the PP was demarcated (that is, visible as a 'plate' near the pial surface) due to a bundle of NF^+ ^fibers that ran below a group of Purkinje cell somata to form the PP (Figure 8F, G). This suggests that the change in orientation or 'posture' required for each Purkinje cell to make its axon relatively deeper may underlie the emergence of the PP at the population level.

In E14.5 *reeler *cerebella lacking the PP, a NF^+ ^fiber bundle was observed above the Purkinje cells' somata (just beneath the pial surface; Figure 8H, I), raising the possibility that the lack of a PP is due to a failure to change the orientation or 'posture' of Purkinje cells between E13.5 and E14.5. Indeed, adenovirus-mediated pulse-chase examinations revealed that E10.5-born Purkinje cells in a dorsal region of E14.5 *reeler *cerebella remained parallel to the pial surface without showing a posture change (Figure 8J, K). While about 40% of the total E10.5-labeled Purkinje cells in normal cerebella (n = 71) were perpendicular to the pial surface (Figure 8E), none of the E10.5-born Purkinje cells adenovirally examined in E14.5 *reeler *cerebella (0/32 cells) showed the perpendicular orientation seen in normal cerebella.

**Figure 9:**
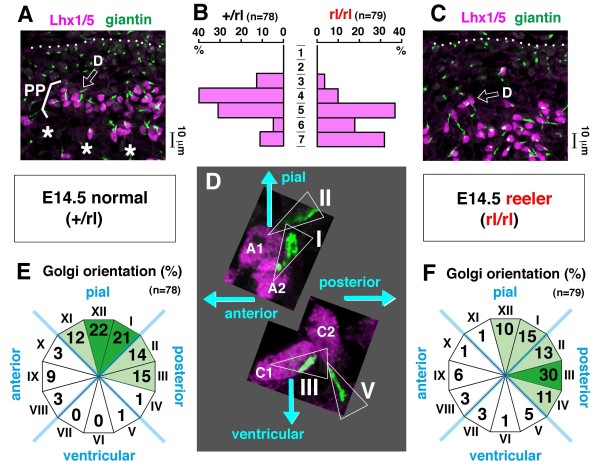
**Orientation of the Golgi apparatus in Purkinje cells of E14.5 normal and *reeler *mice**. **(A, C) **Purkinje cells initiating the formation of the Purkinje plate (PP) in an outer region of normal cerebella (A) and those accumulating in the corresponding region of *reeler *cerebella without forming the PP (C) were analyzed for their Golgi orientation using anti-giantin and anti-Lhx1/5 immunostaining. **(B) **Graphs depicting the relative position of Lhx1/5^+ ^nuclei examined within a range of 70 μm from the pial surface (in increments of 10 μm), showing the existence of a cell-sparse zone (bins 6 and 7; asterisks in (A)) that demarcates pially arranged nuclei (bins 3 to 5) as a plate in +/rl cerebella. **(D-F) **Golgi orientation relative to the nucleus (cells A1 and A2 from (A); cells C1 and C2 from (C)), with the results summarized in (E) (normal cerebella) and (F) (*reeler *cerebella). Golgi orientation towards the pial side (from 'XI o'clock' to 'I o'clock') was observed more frequently in the normal group than in the *reeler *group (Chi-square test, *P *< 0.001). In *reeler *cerebella, Golgi orientation was more posterior than in normal cerebella (Chi-square test, *P *< 0.01). Cells were counted in four independent sections from two embryos in each group (+/rl or rl/rl).

To further compare the morphology of Purkinje cells between normal and *reeler *cerebella, we focused on the orientation of the Golgi apparatus. The Golgi apparatus has recently been used for evaluating whether post-migratory neocortical neurons are oriented properly, with Golgi-containing dendrite-like processes extended towards the pial side [36,37]. We performed immunostaining using anti-giantin to visualize the Golgi. The Purkinje cells beginning to form the PP in an outer region of normal E14.5 cerebella showed a predominant Golgi orientation towards the pial surface, while Purkinje cells that failed to form a clear plate in *reeler *cerebella showed a significantly more posterior Golgi orientation (Figure 9).

**Figure 10:**
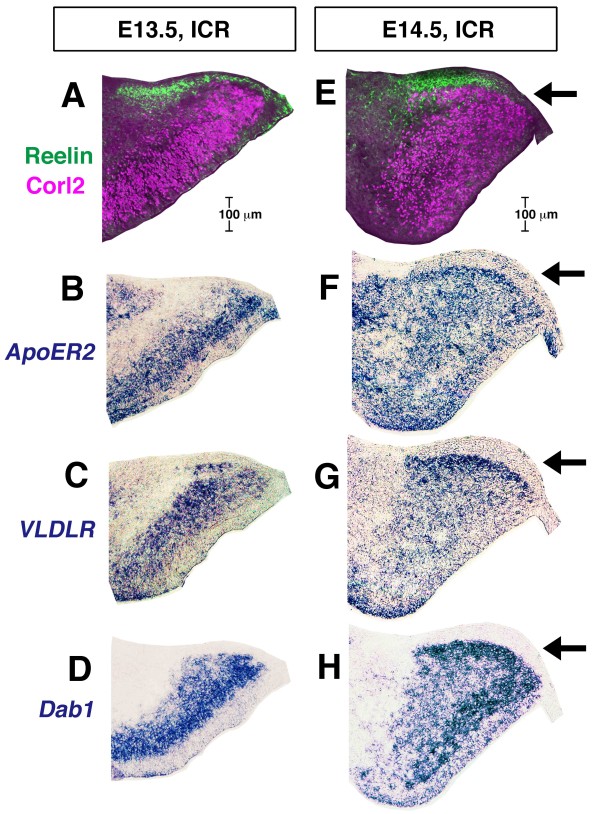
**Spatiotemporal relationship between Purkinje plate formation and Reelin expression**. **(A-H) **Comparison of the distribution of Corl2-expressing Purkinje cells (magenta) with that of Reelin (CR50; green) (A, E), *ApoER2 *mRNA (B, F), *VLDLR *mRNA (C, G), or *mDab1 *mRNA (D, H) in normal cerebella at E13.5 (A-D) and E14.5 (E-H). Purkinje cells just prior to initiating the formation of the PP, are clearly positive for *VLDLR *and *mDab1 *(and also for *ApoER2 *weakly) at E13.5, and the PP (arrow) emerges beneath the Reelin-rich zone (green), with intense signals for *ApoER2*, *VLDLR*, and *mDab1 *at E14.5.

We further examined the spatiotemporal relationship between the posture-change behavior of the early/posterior-born Purkinje cells in initiating the PP and the expression of Reelin, its receptors (apolipoprotein E receptor 2 (ApoER2) and very low-density lipoprotein receptor (VLDLR)) [38], and the adaptor protein mDab1 [39]. At E13.5, Reelin^+ ^cells (presumptive DN neurons) were enriched along the pial surface, covering a dorsal cerebellar region where tangentially oriented Purkinje cells are contained (Figure 10A). The expression of VLDLR and ApoER2 and that of mDab1 were detected throughout the area of Purkinje cells (Corl2^+^) in E13.5 cerebella, including the dorsal region to be used for PP initiation (Figure 10C-E). At E14.5, the PP emerged just below the Reelin-rich zone (Figure 10E), and strong signals for ApoER2, VLDLR, and mDab1 were detected in the PP (Figure 10F-H).

**Figure 11:**
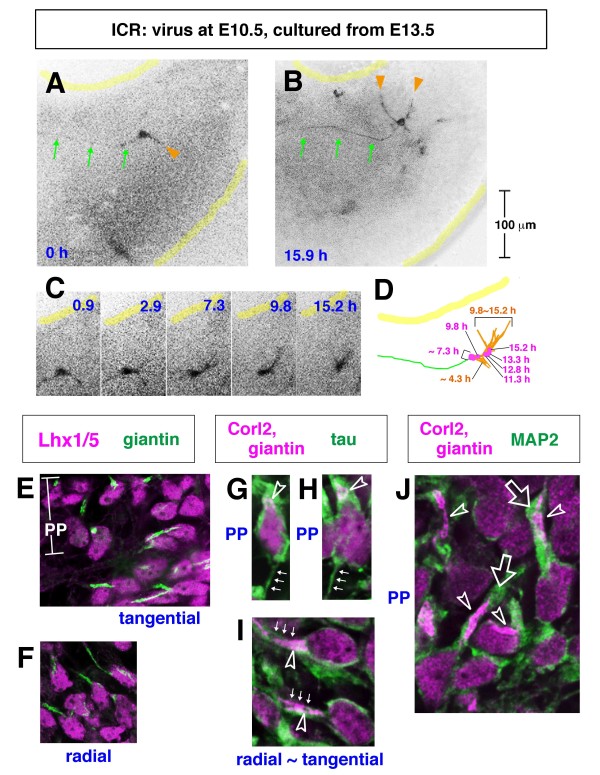
**Purkinje plate formation through Purkinje cells remodeling dendrite-like processes towards the pial side in association with a change in Golgi distribution**. **(A-D) **Time-lapse observation of an E10.5-born Purkinje-like cell in an E13.5 normal cerebellar slice (case presented in Additional file 6). Continuous imaging was carried out automatically (using a 10× objective lens) until the end of culture (15.9 h) (B), when the entire structure of this cell was obtained by reconstructing images captured manually at a higher magnification (20×). Initially (0 h) (A), the labeled cell is horizontally oriented with a long axon-like fiber anteriorly (arrows) and a short, slightly thicker process posteriorly (orange arrowhead). During culture, the cell changes its orientation through extension of new processes towards the pial side (arrowheads in (B)). Note that the remodeling of non-axon-like processes from a posterior or ventricular direction to a more pial direction (B) (orange traces in (D)) is accompanied by a somal movement in the same direction (magenta traces in (D)). **(E-J) **Double or triple immunostaining to depict the position of Golgi in Purkinje cells before and after the formation of the Purkinje plate (PP) *in vivo*. After forming the PP, Purkinje cells (G, H, J, and upper part of E) had Golgi distributed towards the pial side within dendrite-like processes positive for microtubule-associated protein 2 (MAP2) (J). Prior to forming the PP, Purkinje cells, which oriented either radially (F) or tangentially (I, and lower part of E), had Golgi distributed in a proximal part of the thick axon-like process (tau^+^) (I). Axon-like fibers (arrows) are thinner in the PP cells (G, H) than in tangentially oriented cells (I). Open arrowheads indicate the Golgi apparatus (green in (E, F) and magenta in (G-J)). Open thick arrows indicate dendrite-like processes.

To further inquire into how Purkinje cells change their posture under the Reelin-rich zone in E13.5 cerebella, we carried out time-lapse observations. A representative case of single GFP-labeled Purkinje-like cells (monitored for about 16 h from slice preparation at E13.5 following adenovirus injection at E10.5; Figure 11A-D; Additional file 6) directly showed a posture-change behavior as suggested by *in vivo *examination. While an axon-like anteriorly extended fiber remained stable, non-axon (dendrite-like) processes were dynamically remodeled and extended towards the pial side. This remodeling of processes was coupled with somal movement to the pial side, which made the axon-like fiber relatively deeper than the soma.

To better describe this posture-change behavior as a distinct histogenetic step for migratory Purkinje cells, we compared the position of the Golgi apparatus between the most developmentally advanced Purkinje cells that had been incorporated into the PP at E14.5 and younger Purkinje cells exhibiting tangential or radial orientations of the soma in regions deeper than the PP or in E13.5 cerebella. Interestingly, nascent Purkinje cells before and after the formation of the PP distributed their Golgi completely differently (Figure 11E-J). In Purkinje cells displaying morphologies consistent with radial and tangential migration, the Golgi was located in a proximal part of the pially and anteriorly extending axon-like (tau^+^) fibers. In Purkinje cells from the PP, however, the Golgi was found in the dendrite-like process that was positive for microtubule-associated protein 2 (MAP2) and extended towards the pial side. It is likely, therefore, that the Golgi apparatus was excluded from the axon-like part of Purkinje cells and moved into the non-axon part during a transition from the tangentially oriented phase into the PP-forming step.

We carried out an additional three examinations to find out whether factors other than a Reelin-enriched superficial region and Purkinje cell polarization might also be primarily involved in the emergence of the PP. First, migration of RL-derived cells, which produce Reelin without expressing Reelin receptors and mDab1 (Figure 10A-E), was normal in *reeler *cerebella at least until E15.5 (as seen for DN cells positive for Tbr1 in Figure 8G, I and EGL cells positive for Pax6 in Additional file 7D, E, N, O). Second, we found that a portion of accumulating NF^+ ^fibers that demarcate the PP in normal E14.5 cerebella did not contain afferent fibers that were labeled with DiI inserted into the cerebellar peduncular region (Additional file 8A). Third, Nestin^+ ^fibers were radially oriented in *reeler *cerebella at E14.5 (Additional file 8B) in a pattern indistinguishable from that observed in normal E14.5 cerebella (Additional file 7A). These examinations suggest that migration of RL-derived cells, afferent fibers, and radial fiber morphology may not account for the differential Purkinje cell behaviors between normal and *reeler *cerebella during the E13.5 to 14.5 period.

**Figure 12:**
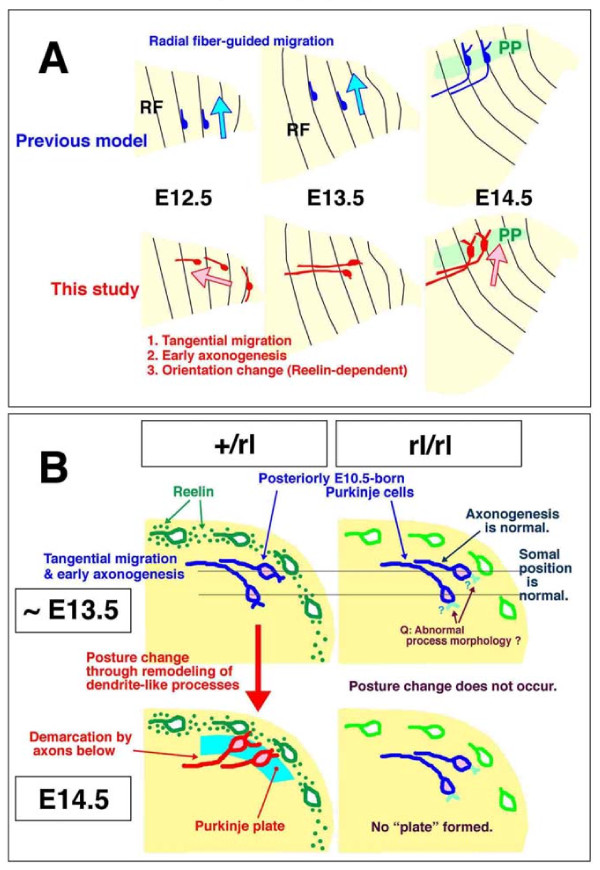
**Schematic illustrations summarizing the present findings**. **(A) **Comparison between the previous model and the present findings of the behavior of nascent Purkinje cells to initiate the PP. Sagittally sectioned cerebella are illustrated and radial fibers (RF) indicated. The pial side is top and posterior is right. **(B) **Illustration showing the differential behaviors of posteriorly E10.5-born Purkinje cells in normal and *reeler *cerebella from E13.5 to E14.5. The most important difference is whether they change their posture/orientation from horizontal or axon-top to axon-bottom (normal) or not (*reeler*). The initial migration (mostly tangential) and axonogenesis (anteriorly) are normal in *reeler *cerebella until E13.5, although Purkinje cells in E13.5 *reeler c*erebella might have slight morphological abnormalities concerning the elaboration of non-axon (dendrite-like) processes on the posterior/ventricular pole of the soma (indicated by question marks).

Taken together, these results suggest that the normal emergence of the PP at E14.5 between the EGL and a cell-sparse axon-rich zone is explained by a Reelin-dependent posture change of early/posterior-born Purkinje cells from a tangential orientation to a more perpendicular orientation to the pial surface. This change in posture occurs through movement of their somata and short dendrite-like processes towards a Reelin-rich zone near the pial surface. Their own axon-like fibers are thus left deeper in the subcortical region (Figure 12A), thereby demarcating their somata as a plate above (Figure 12B). Early axonogenesis is therefore an important means for Purkinje cells to initiate the formation of a discrete layer.

## Discussion

The present study unveiled three important behaviors of nascent Purkinje cells derived from the posterior lateral cerebellum at E10.5: tangential migration, early axonogenesis, and Reelin-dependent 'posture' change to initiate PP formation. This early/posterior-born subset of Purkinje cells is the main contributor to the initiation of PP at E14.5 (Figure 1), and is a good model to investigate the mechanism through which Purkinje cells can be aligned under the regulation of Reelin.

The tangential mode of migration exhibited by these early/posterior-born Purkinje cells is very similar to that of RL-derived prospective DN neurons moving more superficially. We demonstrated immunohistochemically that the non-superficial, tangentially oriented cells were Purkinje cells. The existence of these cells was well recognized in previous Golgi studies, although they were regarded, together with the superficial cells, to be DN neurons [1]. The tangentially oriented Purkinje cells were negative for markers for RL derivatives (Math1/Atoh1, not shown; Pax6 (Additional file 7; Tbr1 (Figure 8)). Not only E10.5-born but also E11.5-born Purkinje cells exhibited tangential morphologies in a posterior part (Additional file 2E). While E10.5-born DN neurons (GFP^+^Lhx1/5^-^) migrated tangentially along the pial surface up to 600 μm from the RL in 2 days, the histogram for the overall E10.5-born Purkinje cells (GFP^+^Lhx1/5^+^) showed a peak at 100 μm from the ventricular surface, with the farthest cells around 160 μm at E12.5 (Figure 1E). This suggests a basic rule for the E10.5 to E12.5 period that neuronal migration velocity is much greater in the tangential mode than in the radial mode. Posterior-born Purkinje cells, which are inherently closer to the pial surface than anterior-born cells, seem to be the quickest to reach an outer zone for subsequent initiation of the PP mainly due to their greater dependence on the tangential mode.

According to the previous model [6,9,23-26], Purkinje cell behavior prior to initiating the PP could be likened to moving from one side across to another along radial fibers (Figure 12A). We propose here that migration of a population of Purkinje cells born early in the posterior part of the cerebellum is more akin to moving along a triangular perimeter (Figure 12A). The former model was coupled with a speculation that the front (outer extremity) of each migratory Purkinje cell may transform into a dendrite whereas an axon will emerge from the rear (inner extremity). Our results show that the origins of the axons and dendrite-like processes in the early/posterior-born Purkinje cells in the lateral cerebellar primordium are opposite of what is expected in the radial migration model. It is currently unclear whether the pially or anteriorly extended processes, which are ahead of outward migrating Purkinje cells, in cerebella at E12.5 can be immediately defined as axons. However, their traceability later during embryonic development along with the overall increase in number and length, as well as the sequential expression of NF from E12.5 (Figure 5) and tau from E13.5 (Figure 6), strongly suggest that they will directly become mature axons. The axonogenic behavior of Purkinje cells resembles that of neurons outside the neocortex; the extension of a prospective axon to the direction of migration (towards the pial/outer side) is suggested in motor neurons in the developing spinal cord [40], retinal ganglion cells [41,42], retinal bipolar cells [43], tectal ganglion cells [44], acoustic nuclei neurons [45], and serotonergic neurons in the hindbrain [46]. Mechanisms regulating the elaboration of a new axon-like pial process from a nascent Purkinje cell (Figure 5C-E) are unknown. But the similarity between the course of axonogenesis by nascent Purkinje cells and that of afferent ingrowth reminds us of an intriguing possibility that afferent fibers might influence Purkinje cell behaviors [47]. Whether some of the axon-like fibers are formed through the inheritance of radial fibers from progenitor cells needs to be determined, as we have observed that progenitor cells in cerebella at E11.5 to E12.5 divide at the ventricular surface while keeping their pial processes (data not shown).

At E13.5, Purkinje cells reach an outer area (150 to 200 μm from the ventricular surface) that underlies the most superficial zone. Although this outer area normally becomes covered with Reelin from this point onward (Figure 10A), it cannot be distinguished between normal and *reeler *cerebella (Figure 7). The outermost Purkinje cells, which subsequently exhibit the earliest Reelin-dependent behavior, are horizontally oriented at this time-point (Figure 2). Then, differential posture-change behaviors between normal and *reeler *Purkinje cells are observed by E14.5 (that is, Purkinje cells in normal cerebella change their orientation as if bending upwards, while those in *reeler *cerebella remain laying down; Figures 8 and 12).

This difference is similar to that previously suggested in E14 to E15 cerebral walls [48,49]. In the normal cerebral wall, early-born neurons that have migrated to an outer area are oriented vertically, with the dendrites arising from the outer pole of the soma and the axon from the inner pole. The axons extended from these early neurons demarcate these cells' somata above as a plate, forming the cortical plate [50]. In the corresponding outer zone in *reeler *cerebral walls, many neurons lie horizontally, which is similar to what we observed for Purkinje cells in E14.5 *reeler *cerebella (Figures 8 and 12). The normal bending up-like posture change of the early/posterior-born Purkinje cells enables the axons to become relatively deep and demarcate their somata above as a plate.

This posture change is likely driven by dynamic extension of dendrite-like processes, coupled with repositioning of Golgi, towards the Reelin-rich zone (Figures 9, 11 and 12). Previous co-culture and transplantation experiments showed that Purkinje cells whose somata were aligned along an artificially provided Reelin-rich zone had extended dendrite-like processes into that zone [22]. Recently, involvement of Reelin in the growth of dendrites has been further demonstrated [36,37,51-56]. Taken together, we conclude that the posture change of early/posterior-born Purkinje cells, which drives the PP emergence in the lateral cerebellum, is explained primarily by the action of Reelin immediately above the Purkinje cells during the E13.5 to E14.5 period. Dendritogenesis may be tightly linked with layer formation.

Associated with the extension of the dendrite-like part, the soma moves towards the outer/pial side. The initial somal movement of the early/posterior-born Purkinje cells is oriented towards the anteriorly growing axon-like part (heading away from the posterior ventricular surfaces). The second step of somal movement towards the pial side can be viewed as a reverse or 'switchback'-like behavior (Figure 12), which has been suggested to occur during migration of tectal, acoustic, and facial nuclei neurons [44,45,56]. Although the initial migration step until E13.5 is tangential and considered to be independent of radial fibers, this second step between E13.5 and E14.5 is more radial and thus the radial fibers might be involved in this step. Further studies are required to determine whether the migration of Purkinje cells other than the early/posterior-born population is also biphasic (or multi-phasic). Comparison between Lhx1/5 and Nestin immunostaining patterns (Additional file 7) raises the possibility that Purkinje cell behavior after the emergence of the PP (Figure 1) might be explained by the guidance of their second (or final) phase of migration by radial fibers. This could correspond to what has been proposed previously [6,9,23-26]. As in the E13.5 to E14.5 period, the most intense extracellular expression of Reelin occurs subpially throughout late embryonic development [16]. Whether the short-range mechanism for Reelin's instruction that we propose for the early/posterior-born Purkinje cells (that is, targeting the action of cells near the Reelin-rich zone) in the lateral cerebellum might be applicable to later-arriving Purkinje cells should be examined further.

It is important to understand how the short-range action mechanism might coincide with other stage- or context-dependent mechanisms. These mechanisms include Reelin's 'encouragement' of migrating or premigratory neurons distant from their final arrangement position [57], Reelin's effects on radial fibers [58-62], and the Reelin-independent 'community effect' among Purkinje cells [63]. Transgenic mice artificially expressing *reelin *under the *nestin *promoter, which works within the VZ (the birthplace of Purkinje cells) until mid-embryonic development, showed an apparently normal arrangement of Purkinje cells by the postnatal period [64]. This may lead to an interpretation that Reelin is not necessarily required at the site of Purkinje cell assembly/alignment, and seemingly contradicts the present short-range action model. Nevertheless, the nestin enhancer/promoter is active not only in the VZ (until around E16.5) but also in glial-like cells radially migrating towards the pial side (from E17.5 onward; our unpublished observation using nestin-enhanced GFP mice [65]). Our current observations of the course of Purkinje cell migration inferred from Lhx1/5 immunostaining, the orientation of Nestin^+ ^fibers (Additional file 7), and the movement of cells belonging to the glial cell lineage [24] during the E15.5 to E18.5 period suggests a potential role for glial cells. In *reeler *cerebella, glial cells can successfully migrate to the subpial region [66]. Reelin artificially provided by glial cells migrating from deep regions to subpial portions in the *nestin-reelin *transgenic *reeler *cerebella might have enabled Purkinje cells to approach the subpial position mainly during this late embryonic stage. It would be intriguing to check whether *nestin-reelin *transgenic *reeler *cerebella might not have a normal PP at E14.5.

## Conclusions

By using an adenovirus-mediated pulse-chase method in combination with recently established Purkinje-cell-specific markers and slice culture, the present study focused on the developing mouse lateral cerebellum until E14.5 and unveiled three important behaviors of early/posterior-born Purkinje cells: tangential migration (at approximately E13.5), early axonogenesis (at approximately E12.5), and Reelin-dependent 'posture' change initiating their layer formation (between E13.5 and E14.5). Early axonogenesis by Purkinje cells contributes to their formation of a discrete layer. Our study provides a solid basis for further elucidation of Reelin's function and the mechanisms underlying the cerebellar corticogenesis, and will contribute to the understanding of how polarization of individual cells drives overall brain morphogenesis.

## Methods

### Animals

B6C3Fe-a/a-rl mice (heterozygous for the *reeler *mutation) were obtained from the Jackson Laboratory. Heterozygous (+/*rl*) and homozygous (*rl*/*rl*) embryos were generated by pairing female heterozygotes and male homozygotes and genotyped as described previously [67]. For 'normal' cerebellar specimens, both the heterozygous mice and ICR mice were used. Only homozygous (*rl*/*rl*) mice are called *reeler *in this paper. The day of vaginal plug identification was defined as E0.5.

### Adenovirus

Adenoviral construction was carried out as previously described [27,28]. The adenoviral vector Adex-CAG-Lyn-Venus expresses membrane-targeted Venus under control of the CAG promoter. Lyn-Venus was generated by fusing the sequence of the palmitoylation site of tyrosine kinase Lyn to the amino terminus of Venus [32,33]. Adenoviral vector solution was injected into the fourth ventricle of embryos at each day from E10.5 to E12.5 using a trans-illumination system of *in utero *surgery [68]. For histological examination using frozen sections (16 μm), the titer of adenovirus vector solution was set to a level at which <5 labeled cells in each section could be seen. With this dilution, data used for making the histograms in Figure 1E were obtained from three to seven normal (ICR mouse) embryos at each stage. For live imaging on normal cerebellar slices (200 to 250 μm), viral solution was further diluted to ensure sporadic labeling. For comparisons between *+/rl *and *rl/rl *littermates at E13.5 (Figure 7C), adenovirus-mediated pulse-chase examination was carried out using a more concentrated viral solution.

### Immunohistochemistry

Embryos at each day from E13.5 to E18.5 were perfused transcardially with periodate-lysine-paraformaldehyde (PLP) fixative [69]. Younger (E11.5 and E12.5) embryos were fixed by immersion in PLP at 4°C for 2 h. Isolated brains were postfixed in PLP at 4°C for 1 to 2 h, immersed in 20% sucrose, embedded in OCT compound (Miles, Elkhart, IN, USA), and then frozen and sectioned sagittally and in some cases coronally (16 μm). Frozen sections were treated with the following primary antibodies: anti-Lhx1/5 (Lim1/2; mouse, Hybridoma Bank [4F2]); anti-Corl2 (rabbit) [30]; anti-Nestin (rabbit, gift from Dr Yasuhiro Tomooka) [70]; RC2 (mouse IgM, gift from Dr Miyuki Mamamoto) [71]; anti-Neurofilament (mouse, Hybridoma Bank [2H3]; rabbit, BIOMOL, Plymouth Meeting, PA, USA); anti-GFP (rat, Nakarai Tesque, Inc., Kyoto, Japan; rabbit, MBL, Nagoya, Japan, anti-Tbr1 (rabbit, Abcam, Tokyo, Japan), anti-Reelin (mouse [CR50]) [14,16]; Pax6 (rabbit, Covance, Berkeley, CA, USA); anti-Math1 (Hybridoma Bank); anti-tau (mouse, Chemicon, Billerica, MA, USA); anti-MAP2 (mouse, Sigma, St. Louis, MO, USA); and anti-giantin (rabbit, Covance). For immunostaining of live DiI-labeled cells in cerebellar slices, vibratome sections (50 μm) made after the slices were fixed with 4% paraformaldehyde at 4°C for 20 minutes were treated with antibodies at 4°C overnight.

### Slice culture

Cerebellar primordia were isolated from E12.5 or E13.5 embryos that had been infected *in utero *with GFP-adenovirus at E10.5 and sliced using fine microknives, as previously described for cerebral wall slices [31,32]. Because the long axis of each cerebellar primordium is roughly perpendicular to the rostorocaudal (anteroposterior) axis of each embryo and bridges both sides of the metencephalon, the plane of each live slice (200 to 250 μm thick, 6 to 8 slices per embryo) was made by sectioning almost perpendicular to the long axis of the isolated primordium and was generally close to the *in vivo *sagittal plane. In a lateral region, where the cerebellar primordium is more oblique *in vivo *(less perpendicular to the embryo's long axis), the plane of the live slices was less sagittal. The slices were then transferred to a glass-bottom 35 mm plastic culture dish containing enriched culture medium, a 1:1 mixture of DMEM and Ham's F-12 (phenol red-free) supplemented with insulin (25 μg/ml), transferrin (100 μg/ml), progesterone (20 nM), sodium selenate (30 nM, Sigma), putrescine (60 μM), epidermal growth factor (10 ng/ml), basic fibroblast growth factor (10 ng/ml), horse serum (5%), and fetal calf serum (5%). Up to ten slices were then transferred with 100 to 150 μl of the enriched medium to the center of the dish, then mixed with 100 to 150 μl of 2× type I collagen gel solution (Cellmatrix IA, Nitta Gelatin, Tokyo, Japan; previously diluted to 0.5 to 0.6 mg/ml with distilled water, 5× phenol red-free DMEM, and a neutralizing buffer according to the manufacturer's protocol, and kept on ice until use). The gel (final concentration 0.25 to 0.3 mg/ml) was immediately spread onto the dish base using a pipette tip. After nearly allowing the gel to solidify (about 5 minutes) with the ventricular and pial surfaces of the slices perpendicular to the dish base (arranged with a tungsten needle), the dish was placed in an incubator (37°C, 40% O_2 _and 5% CO_2 _) (Astec, Kasuya, Fukuoka, Japan) to allow further gel solidification (5 to 10 minutes). The enriched medium (0.8 ml) was dropped onto the gel and spread over the entire dish surface.

### Imaging

For on-stage culture, an inverted epifluorescence microscope (IX70, Olympus) equipped with a 10× objective lens (UPlanF1; numerical aperture = 0.30) was used. In this system, slices were continuously cultured in a chamber providing a humidified atmosphere of 5% CO_2 _and 40% O_2 _(Tokken, Kashiwa, Chiba, Japan). Imaging was performed at 5-minute intervals using a cooled charge-coupled device (CCD) camera (CoolsnapHQ, Roper Scientific, Tucson, AZ, USA), which was automatically controlled by METAMORPH 6.3r-1 software (Molecular Devices, Silicon Vally, CA, USA) as described previously [72]. Some live images of Venus-labeled cells were captured manually using a different system composed of an inverted epifluorescence microscope (IX71, Olympus) equipped with a 20× objective lens (UPlanApo; numerical aperture = 0.70) and a cooled CCD camera (ORCA-ER, Hamamatsu Photonics, Hamamatsu, Shizuoka, Japan) and IPLab 3.6 software (Scanalytics, Inc., Rockville, MD, USA). The same manual capturing system was used for most of the presented immunofluorescence images. For some of the immunostained specimens, confocal microscopy was performed using a Yokogawa CSU10 (40×, 100×) (Tokyo, Japan) and an Olympus FV1000 (40×) (Tokyo, Japan).

### DiI labeling

For single cell-level visualization within cerebellar primordia, fine crystals (< 30 μm) of DiI C18(3) (1,1-dioctadecyl-3,3,3,3-tetramethylindocarbocyanine perchlorate; D-282, Molecular Probes, Eugene, OR, USA) were inserted into live cerebella from the pial surface using a small make-up brush, and slices were made and incubated at 37°C (40% O_2 _and 5% CO_2_) for 1 to 2 h. After live identification of DiI-labeled cells, slices were fixed and further processed for immunostaining. For labeling of afferent fibers from the vestibular ganglion and those of the cerebellar peduncle, crystals of DiI were inserted into fixed brains using forceps. The DiI-inserted brains were incubated at 37°C for several days, and then vibratome sectioned.

### *In situ *hybridization

Probes to determine the expression of *vldlr*, *apoER2*, and *mDab1 *were designed according to Trommsdorff *et al*. [38]. Cerebella were fixed with 4% paraformaldehyde at 4°C overnight. Frozen sections (16 μm) were pretreated with 4% paraformaldehyde (5 minutes), 0.1 M triethanolamine/0.25% acetic anhydride (10 minutes), and hybridized at 60°C for 15 h in a solution containing 50% formamide, 0.1% SDS, 0.64 mg/ml tRNA, and 0.5 μg/ml riboprobe.

## Abbreviations

APOER2: apolipoprotein E receptor 2; DMEM: Dulbecco's modified Eagle's medium; DII: 1,1'-dioctadecyl-3,3,3',3'-tetramethylindocarbocyanine perchlorate; DN: deep nuclear; E: embryonic day; EGL: external granular layer; GFP: green fluorescent protein; NF: Neurofilament; PLP: periodate-lysine-paraformaldehyde; PP: Purkinje plate; RL: rhombic lip; VLDLR: very low-density lipoprotein receptor; VZ: ventricular zone.

## Competing interests

The authors declare that they have no competing interests.

## Authors' contributions

TM designed the study, carried out virus labeling experiments and culture, analyzed the data, and wrote the paper. YO contributed to molecular identification of cells. MOk and AK contributed to *in situ *hybridization. MH participated in adenoviral and DiI experiments. MM, AS, and MOg contributed to live and immunohistochemical examinations. YO, AS, AK, MH, and MOg provided intellectual guidance. All authors read and approved the final manuscript.

## Supplementary Material

Additional file 1 **Figure S1: experimental procedures for ***in utero ***adenoviral injection and the analysis of the distribution of nascent Purkinje cells in embryonic cerebella**. The adenoviral injection protocol was based on studies by Hashimoto and Mikoshiba [27,28] that showed that almost all Purkinje cells were labeled by daily injections from E10.5 to E12.5. The histogram in Figure 1 was made through repeated measurements of the distance from the nucleus/soma of each GFP and Lhx1/5 double positive nascent Purkinje cell to the ventricular surface. Click here for file

Additional file 2 **Figure S2: radially and tangentially elongated nascent Purkinje cells in E12.5 and E13.5 normal cerebella**. **(A-D) **E10.5-adenovirally labeled Lhx1/5^+ ^cells. Their traces are included in Figure 2I, L. In (A), cell *a *is tangentially oriented (resembling case 1a of Figure 2) while cell *b *is radially oriented near the RL (similar to case 2 of Figure 2). In (C), one of the two GFP^+^Lhx1/5^+ ^cells whose somata are on the outer border of the VZ has a long pial process (cell *a*) while the other cell does not. Of the two pially elongated nascent Purkinje cells in (C), one cell (*b*) has a ventricular process while the other cell (*a*) does not, suggesting that the departure of nascent Purkinje cells from the VZ may proceed through the disappearance of the ventricular process, as is the case for daughter cells in the developing cerebral wall. In (D), a Purkinje cell extending an axon-like process anteriorly (arrowed; resembling case 4 of Figure 2) is shown. **(E) **An E11.5-born Purkinje cell exhibiting tangential morphology in an E13.5 cerebellum. **(F) **DiI-based visualization of radial fibers extending from nascent Purkinje cells in an E12.5 cerebellum. Fine DiI crystals were inserted into an outer cerebellar region corresponding to an area enriched with Neurofilament^+ ^fiber bundles (Figure 2K and Additional file 4A) using fine make-up brushes. Anti-Lhx1/5 immunostaining of sagittal vibratome sections (50 μm thick) revealed that the indicated three cells having a DiI^+ ^pial process are positive for Lhx1/5, which is similar to the case in (B). Click here for file

Additional file 3 **Supplemental Movie 1: migration of an E10.5-born Purkinje-like cell in an E12.5 cerebellar slice**. The labeled cell (of constant 150 μm length) exhibits somal (green arrow) movement away from the ventricular surface in a fashion that can be considered radial migration, but its leading process (magenta arrow at its tip) moves tangentially (parallel to the pial surface). Figure 3A-D are snapshots taken from this movie. Generally, slices prepared from E12.5 cerebella tend to bend during culture, becoming more 'V' or 'U' shaped than when *in vivo*, which sometimes results in partial detachment of slices from collagen gel, as in this case. Click here for file

Additional file 4 **Supplemental Figure 3: early axonogenesis by nascent Purkinje cells**. **(A) **Double immunostained cerebellar sections showing that nascent Purkinje cells in E12.5 cerebella (stained with anti-Lhx1/5) express Neurofilament. **(B) **Time-lapse observation of the extension of an axon-like fiber by an E10.5-born Purkinje-like cell in an E12.5 cerebellar slice. A cone-like growth at the distal tip (red arrow) moves anteriorly while the soma stays at the same position. **(C) **The earliest afferent fibers from the vestibular ganglion do not run radially within E12.5 cerebella. Although a huge bundle of DiI^+ ^afferent fibers derived from the vestibular gangion is observed in an outer area of sagittal cerebellar slices, a deeper cerebellar region containing many radially oriented Neurofilament^+ ^fibers as illustrated in Figures 2K, 4A and panel (A) here does not show DiI-labeled fibers, suggesting that the radial Neurofilament^+ ^fibers are mostly efferent fibers originating from nascent Purkinje cells. Click here for file

Additional file 5 **Supplemental Movie 2: axonogenic behavior of an E10.5-born Purkinje-like cell in an E13.5 cerebellar slice**. While the soma (green arrow) does not move, the tip of the process (magenta arrow) is seen moving anteriorly. Figure 4C-E are snapshots taken from this movie case. Click here for file

Additional file 6 **Supplemental Movie 3: 'posture' change exhibited by an E10.5-born Purkinje-like cell in an E13.5 cerebellar slice**. Initially, the cell's orientation is almost completely horizontal and tangential (parallel to the pial surface), with an axon-like process anteriorly (green arrows) and a shorter process posteriorly and ventricularly (orange arrow). During culture, the non-axon-like process is dynamically remodeled and changes its direction towards the pial surface, which is accompanied by movement of the soma (magenta arrow) in the same direction. Although the axon-like part is not fully focused at the end of culture and not so clearly seen in this movie, the maintenance of this part anteriorly is confirmed by a separate observation carried out manually using a different microscope system (Figure 7F-I). Click here for file

Additional file 7 **Supplemental Figure 4: Stage-dependent changes of the expression of Nestin, Pax6, and Lhx1/5 in normal and *reeler *cerebella from E14.5 to E18.5**. **(A-O) **Stage-dependent changes of the expression of Nestin (A-C), Pax6 (D, E, N, O), and Lhx1/5 (F-M) in normal (A-I) and *reeler *(J-O) cerebella from E14.5 to E18.5. At E14.5, when the presence (normal) or absence (*reeler*) of the PP is evident (asterisk), the degree of anterior spreading of Pax6^+ ^EGL cells is indistinguishable between normal and *reeler *cerebella. Also at E15.5, the anterior spreading of Pax6^+ ^EGL cells seems to be normal in *reeler *cerebella, although there is an abnormal gap between the EGL and Purkinje cells (arrowheads) where some Pax6^+ ^cells are scattered. Normal migratory patterns of Purkinje cells from E14.5 to E18.5 (G-I) (indicated with blue arrows in each panel as a result of migration from the previous stage) have similarity in direction with the orientation of Nestin^+ ^radial fibers (red highlighted in (A-C)). The changes of the flow of migration from dorsal-directed (arrows in (G)) to more multi-directional (arrows in (H, I), both anteriorly and posteriorly diverged flows) appear to be preceded by the modification in the direction of Nestin^+ ^fibers (which showed similar but slightly earlier changes from a dorsal-only directed pattern (A) to a more multi-directional pattern (B, C)). Click here for file

Additional file 8 **Supplemental Figure 5**. **(A) **DiI crystals were inserted into the cerebellar peduncular region to determine whether afferent fibers, and not only efferent fibers, from Purkinje cells might also contribute to the demarcation of the Purkinje plate (PP). In all cases examined (n = 5), the Neurofilament^+ ^fiber-enriched region to demarcate the PP (encircled) was negative for DiI^+ ^afferent fibers. Representative sagittal sectional views from one case are shown. **(B) **Radial fibers are normal in E14.5 *reeler *cerebella. Sagittal sections of an E14 *reeler *cerebellum were simultaneously double immunostained with anti-Lhx1/5 and Nestin. Within the outer region where the lack of the PP is evident (red square) (Figure 1C, D; Additional file 7F, J), Nestin^+ ^fibers run radially, displaying an almost identical pattern compared with that in normal E14.5 cerebella (Additional file 7A). This result was reproduced using different E14.5 *reeler *cerebella (n = 4). Click here for file
