# Supplementary figures and images for: Migration, early axonogenesis, and Reelin-dependent layer-forming behavior of early/posterior-born Purkinje cells in the developing mouse lateral cerebellum
Source: Neural Dev. 2010 Sep 1;5:23. doi: 10.1186/1749-8104-5-23 (PMC2942860; doi:10.1186/1749-8104-5-23)

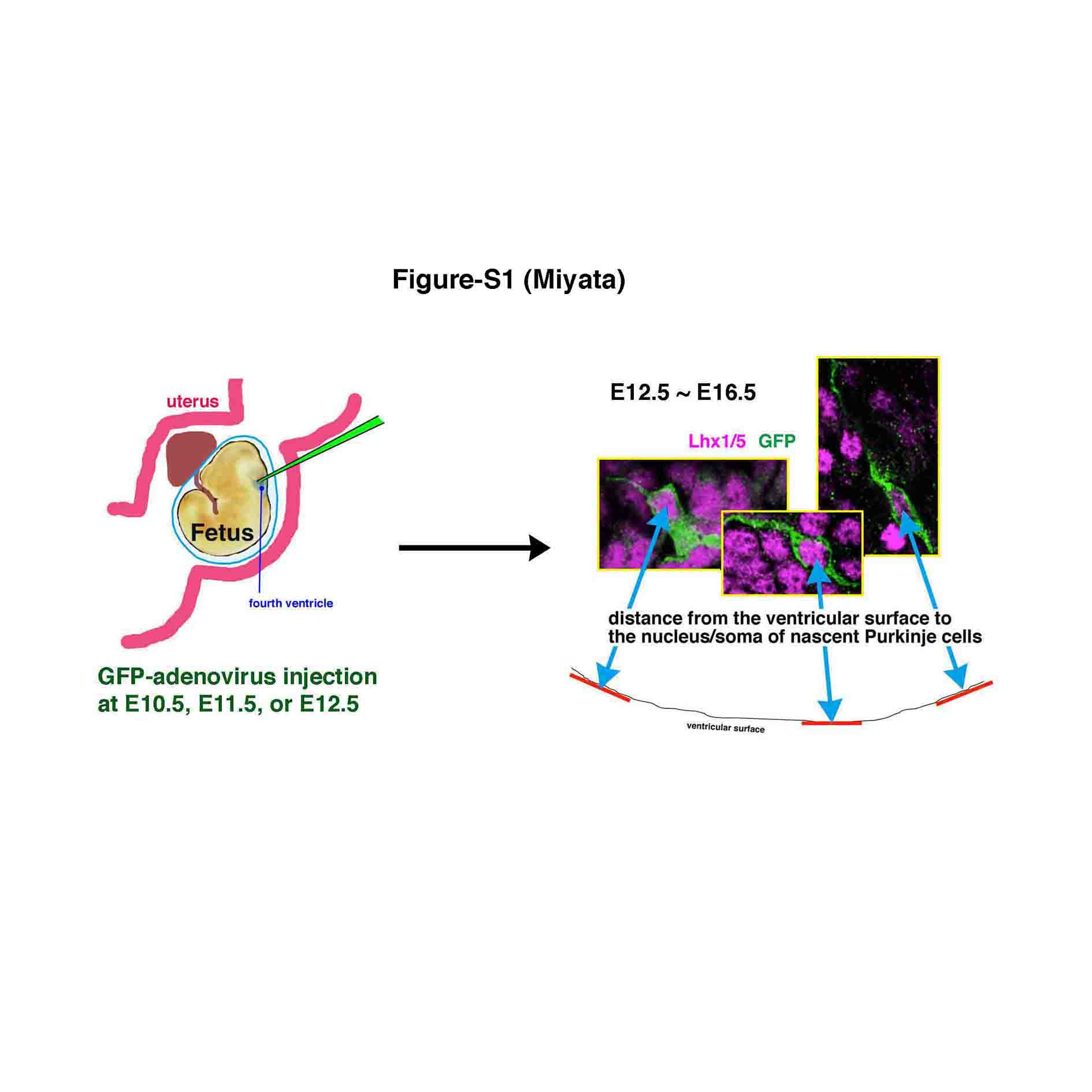

Supplement: Additional file 1 — Figure S1: experimental procedures for in utero adenoviral injection and the analysis of the distribution of nascent Purkinje cells in embryonic cerebella. The adenoviral injection protocol was based on studies by Hashimoto and Mikoshiba [27,28] that showed that almost all Purkinje cells were labeled by daily injections from E10.5 to E12.5. The histogram in Figure 1 was made through repeated measurements of the distance from the nucleus/soma of each GFP and Lhx1/5 double positive nascent Purkinje cell to the ventricular surface. [file 1749-8104-5-23-S1.JPEG]

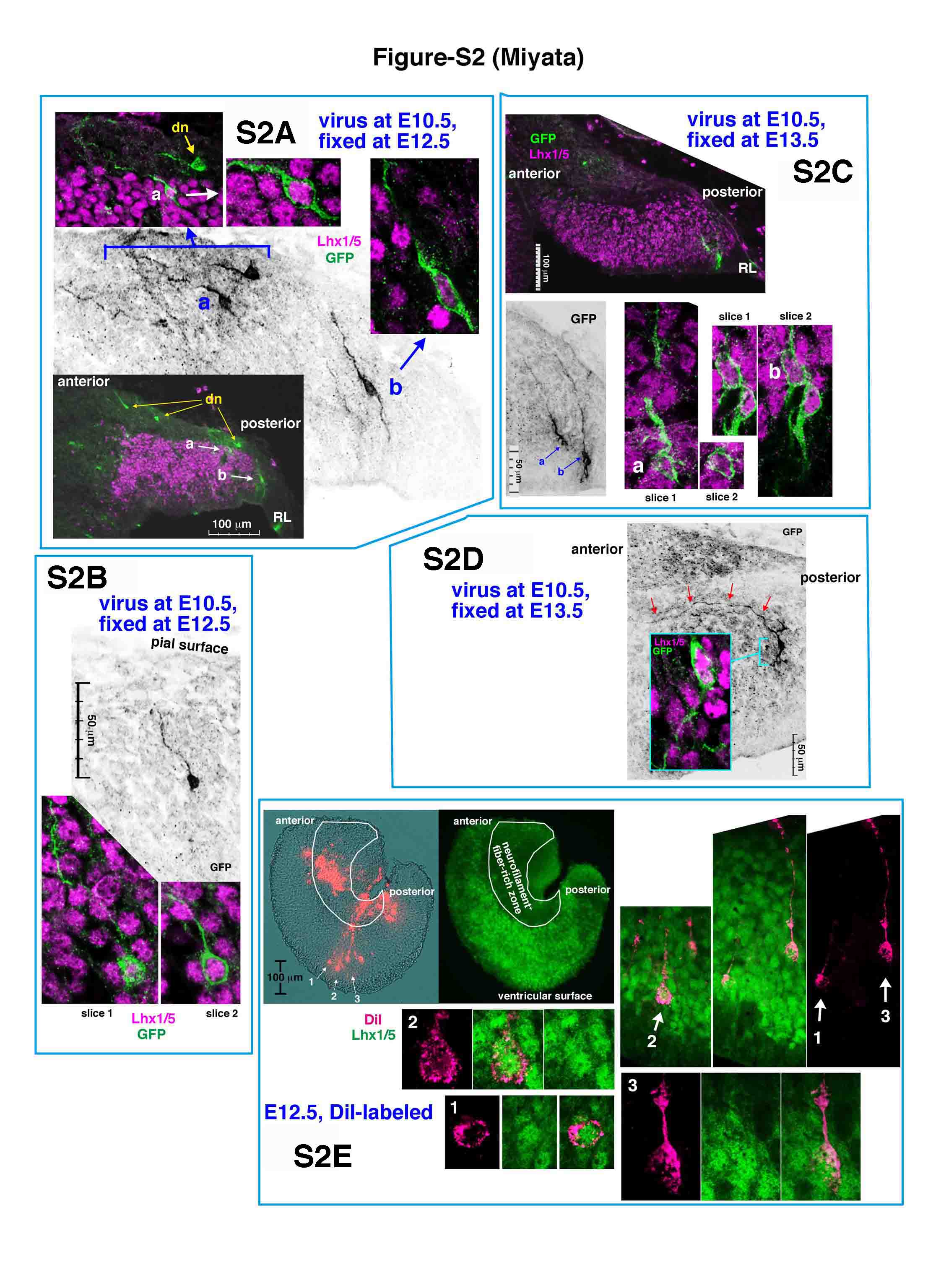

Supplement: Additional file 2 — Figure S2: radially and tangentially elongated nascent Purkinje cells in E12.5 and E13.5 normal cerebella. (A-D) E10.5-adenovirally labeled Lhx1/5+ cells. Their traces are included in Figure 2I, L. In (A), cell a is tangentially oriented (resembling case 1a of Figure 2) while cell b is radially oriented near the RL (similar to case 2 of Figure 2). In (C), one of the two GFP+Lhx1/5+ cells whose somata are on the outer border of the VZ has a long pial process (cell a) while the other cell does not. Of the two pially elongated nascent Purkinje cells in (C), one cell (b) has a ventricular process while the other cell (a) does not, suggesting that the departure of nascent Purkinje cells from the VZ may proceed through the disappearance of the ventricular process, as is the case for daughter cells in the developing cerebral wall. In (D), a Purkinje cell extending an axon-like process anteriorly (arrowed; resembling case 4 of Figure 2) is shown. (E) An E11.5-born Purkinje cell exhibiting tangential morphology in an E13.5 cerebellum. (F) DiI-based visualization of radial fibers extending from nascent Purkinje cells in an E12.5 cerebellum. Fine DiI crystals were inserted into an outer cerebellar region corresponding to an area enriched with Neurofilament+ fiber bundles (Figure 2K and Additional file 4A) using fine make-up brushes. Anti-Lhx1/5 immunostaining of sagittal vibratome sections (50 μm thick) revealed that the indicated three cells having a DiI+ pial process are positive for Lhx1/5, which is similar to the case in (B). [file 1749-8104-5-23-S2.JPEG]

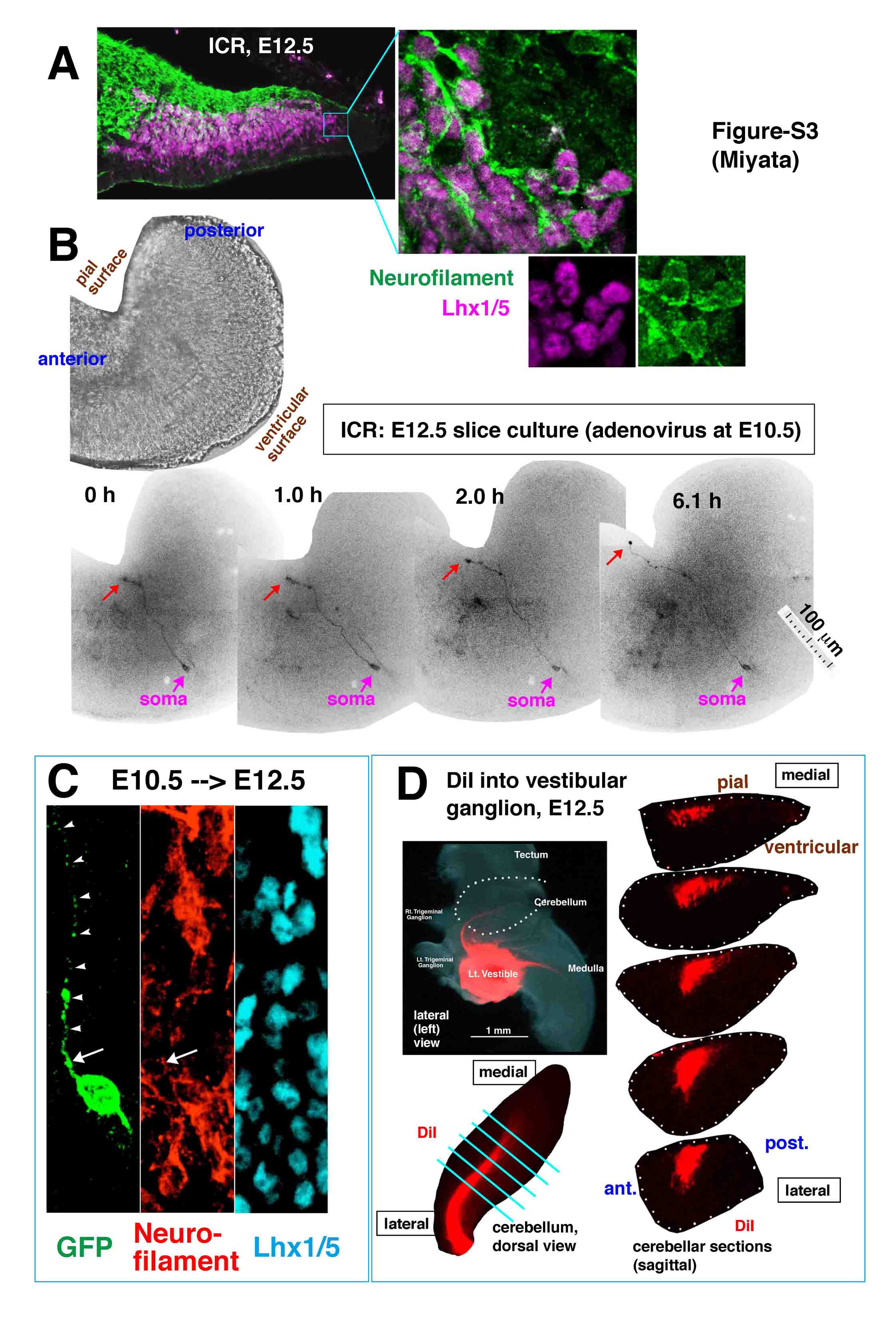

Supplement: Additional file 4 — Supplemental Figure 3: early axonogenesis by nascent Purkinje cells. (A) Double immunostained cerebellar sections showing that nascent Purkinje cells in E12.5 cerebella (stained with anti-Lhx1/5) express Neurofilament. (B) Time-lapse observation of the extension of an axon-like fiber by an E10.5-born Purkinje-like cell in an E12.5 cerebellar slice. A cone-like growth at the distal tip (red arrow) moves anteriorly while the soma stays at the same position. (C) The earliest afferent fibers from the vestibular ganglion do not run radially within E12.5 cerebella. Although a huge bundle of DiI+ afferent fibers derived from the vestibular gangion is observed in an outer area of sagittal cerebellar slices, a deeper cerebellar region containing many radially oriented Neurofilament+ fibers as illustrated in Figures 2K, 4A and panel (A) here does not show DiI-labeled fibers, suggesting that the radial Neurofilament+ fibers are mostly efferent fibers originating from nascent Purkinje cells. [file 1749-8104-5-23-S4.JPEG]

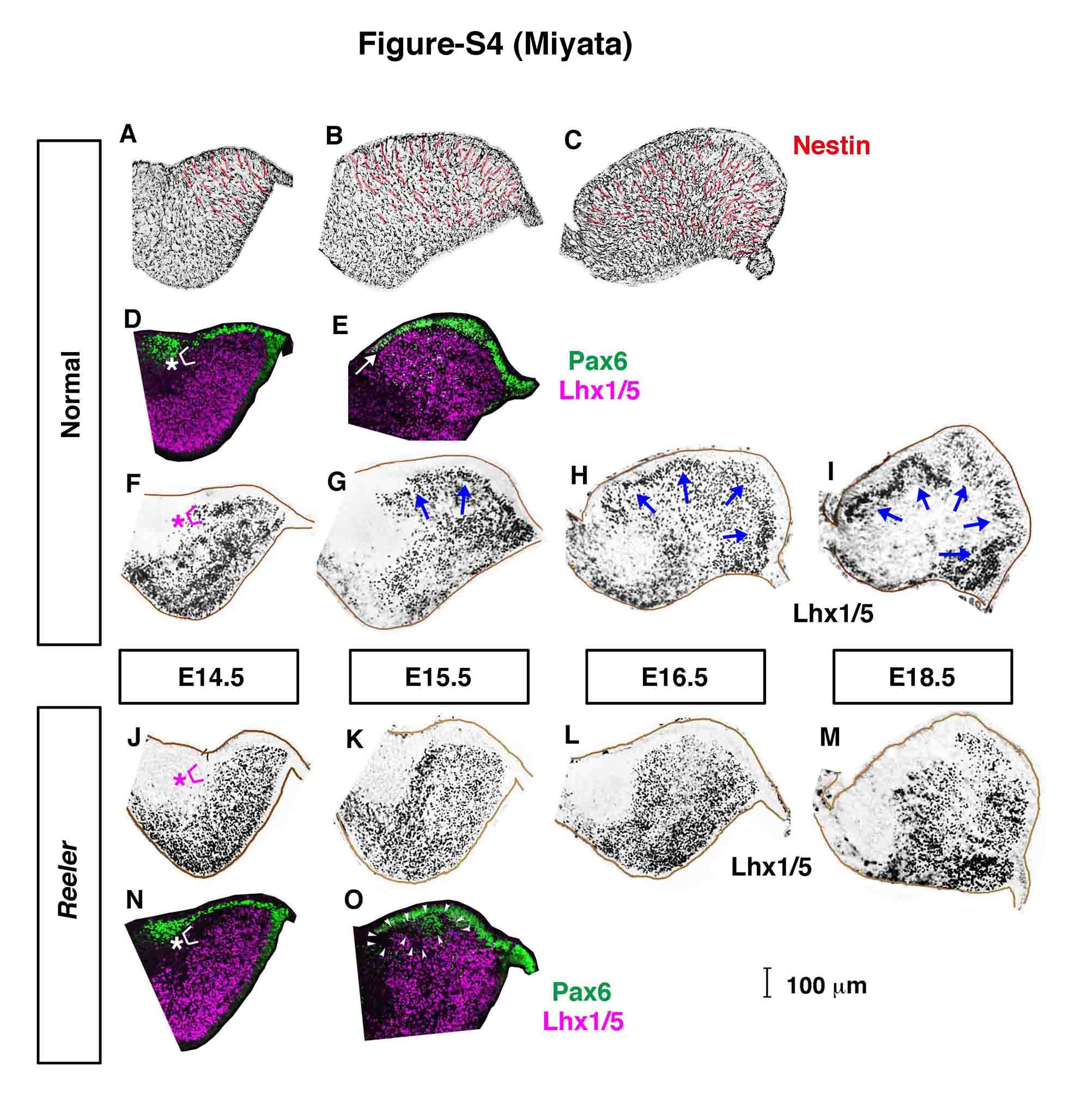

Supplement: Additional file 7 — Supplemental Figure 4: Stage-dependent changes of the expression of Nestin, Pax6, and Lhx1/5 in normal and reeler cerebella from E14.5 to E18.5. (A-O) Stage-dependent changes of the expression of Nestin (A-C), Pax6 (D, E, N, O), and Lhx1/5 (F-M) in normal (A-I) and reeler (J-O) cerebella from E14.5 to E18.5. At E14.5, when the presence (normal) or absence (reeler) of the PP is evident (asterisk), the degree of anterior spreading of Pax6+ EGL cells is indistinguishable between normal and reeler cerebella. Also at E15.5, the anterior spreading of Pax6+ EGL cells seems to be normal in reeler cerebella, although there is an abnormal gap between the EGL and Purkinje cells (arrowheads) where some Pax6+ cells are scattered. Normal migratory patterns of Purkinje cells from E14.5 to E18.5 (G-I) (indicated with blue arrows in each panel as a result of migration from the previous stage) have similarity in direction with the orientation of Nestin+ radial fibers (red highlighted in (A-C)). The changes of the flow of migration from dorsal-directed (arrows in (G)) to more multi-directional (arrows in (H, I), both anteriorly and posteriorly diverged flows) appear to be preceded by the modification in the direction of Nestin+ fibers (which showed similar but slightly earlier changes from a dorsal-only directed pattern (A) to a more multi-directional pattern (B, C)). [file 1749-8104-5-23-S7.JPEG]

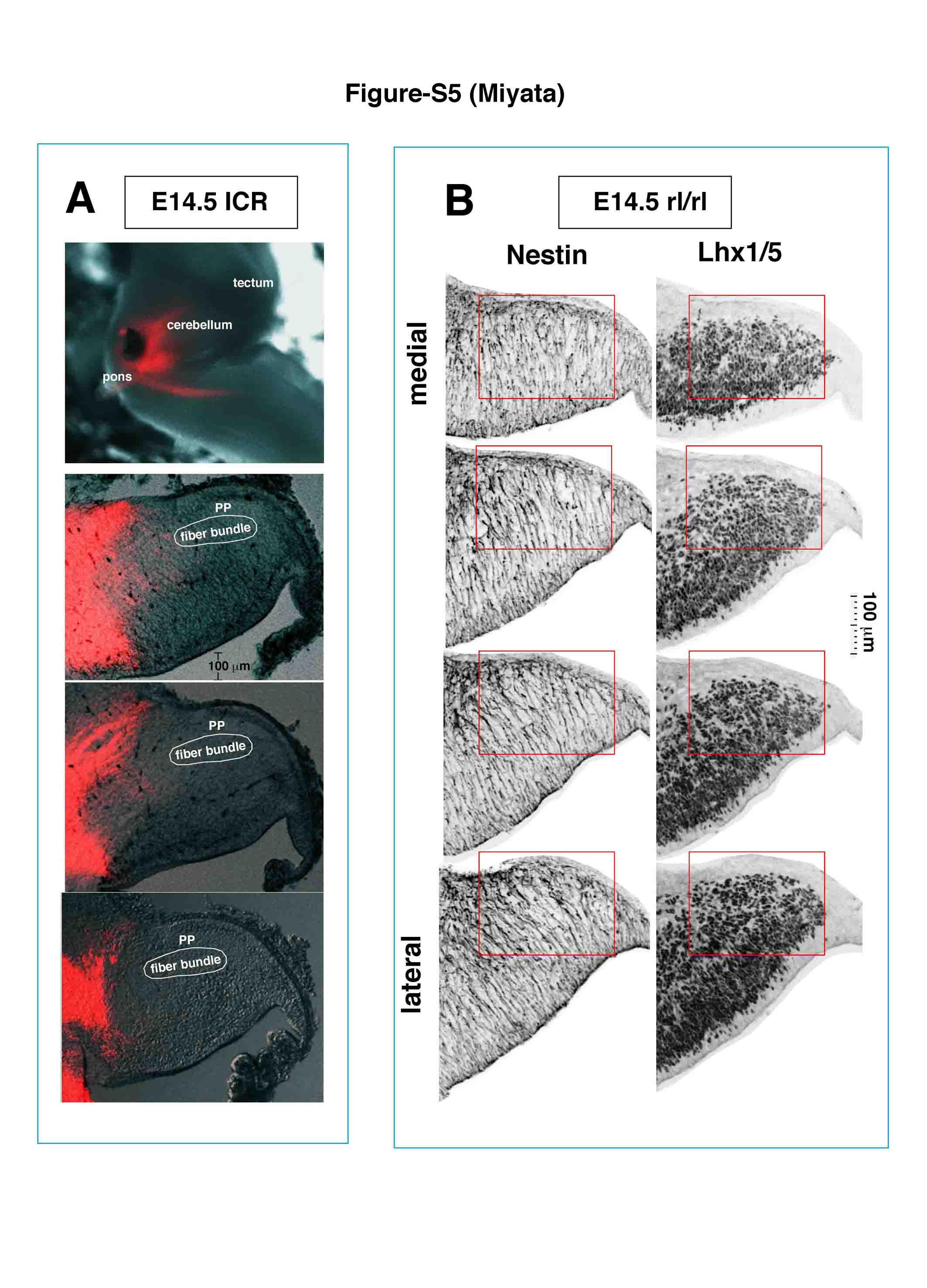

Supplement: Additional file 8 — Supplemental Figure 5. (A) DiI crystals were inserted into the cerebellar peduncular region to determine whether afferent fibers, and not only efferent fibers, from Purkinje cells might also contribute to the demarcation of the Purkinje plate (PP). In all cases examined (n = 5), the Neurofilament+ fiber-enriched region to demarcate the PP (encircled) was negative for DiI+ afferent fibers. Representative sagittal sectional views from one case are shown. (B) Radial fibers are normal in E14.5 reeler cerebella. Sagittal sections of an E14 reeler cerebellum were simultaneously double immunostained with anti-Lhx1/5 and Nestin. Within the outer region where the lack of the PP is evident (red square) (Figure 1C, D; Additional file 7F, J), Nestin+ fibers run radially, displaying an almost identical pattern compared with that in normal E14.5 cerebella (Additional file 7A). This result was reproduced using different E14.5 reeler cerebella (n = 4). [file 1749-8104-5-23-S8.JPEG]
